# A robust intracellular metabolite extraction protocol for human neutrophil metabolic profiling

**DOI:** 10.1371/journal.pone.0209270

**Published:** 2018-12-20

**Authors:** Susama Chokesuwattanaskul, Marie M. Phelan, Steven W. Edwards, Helen L. Wright

**Affiliations:** 1 Biochemistry Department, Institute of Integrative Biology, University of Liverpool, Liverpool, United Kingdom; 2 Faculty of Medicine, Chulalongkorn University, Bangkok, Thailand; 3 HLS Technology Directorate, University of Liverpool, Liverpool, United Kingdom; 4 Department of Musculoskeletal Biology I, Institute of Ageing and Chronic Disease, University of Liverpool, Liverpool, United Kingdom; Korea University, REPUBLIC OF KOREA

## Abstract

Neutrophils are phagocytic innate immune cells that play essential roles in host defence, but are also implicated in inflammatory diseases such as rheumatoid arthritis (RA) where they contribute to systemic inflammation and joint damage. Transcriptomic analysis of neutrophils has revealed significant changes in gene expression in neutrophils activated *in vitro* by cytokines and *in vivo* during inflammation in RA. However, there are no reports on the global metabolomic changes that occur as a consequence of this activation. The aim of this study was to establish protocols for the study of changes in the metabolome of human neutrophils using ^1^H NMR spectroscopy. Sample preparation and spectral analysis protocols were optimised using neutrophils isolated by Ficoll-Paque, with decreased washing steps and inclusion of a heat-shock step to quench metabolite turnover. Cells were incubated ± PMA for 15 min in HEPES-free media and samples were analysed by NMR using a 700 MHz NMR Avance IIIHD Bruker NMR spectrometer equipped with a TCI cryoprobe. Chenomx, Bruker TopSpin and AMIX software were used to process spectra and identify metabolites. Principal Component Analysis (PCA) and signalling pathway analysis was carried out using Metaboanalyst. Cell number and number of scans (NS) were optimised as >3.6 million cells and 512 NS. 327 spectral bins were defined in the neutrophil spectra, of which 287 (87.7%) were assigned to 110 metabolites that included: amino acids, peptides and analogues; carbohydrates, carbonyls and alcohols; nucleotides, nucleosides and analogues; lipids and lipid-like molecules; benzenoids; and other organic compounds. 43 metabolites changed at least 1.5 fold (increase or decrease) after the addition of PMA for 5 or 15 min. Pathway analysis revealed that PMA affected nicotinate and nicotinamide metabolism, aminoacyl-tRNA biosynthesis and glycolysis, suggesting a redirection of glucose metabolism from glycolysis to the pentose phosphate pathway and production of NADPH for activation of the NADPH oxidase and subsequent respiratory burst. We have developed protocols for the study of human neutrophils by ^1^H NMR spectroscopy. Importantly, this methodology has sufficient sensitivity and reproducibility to detect changes in metabolite abundance from cell numbers typically collected from clinical samples or experiments with multiple assay conditions.

## Introduction

Neutrophils are immune cells that form the major arm of innate immunity, killing invading microbes mainly through phagocytosis, granule enzyme activation and production of reactive oxygen species (ROS)[1, 2]. In healthy individuals, neutrophils patrol the peripheral blood and mucous membranes in an inactive or “resting state”, but upon infectious challenge can be rapidly primed by cytokines (such as GM-CSF and TNF) and toll-like receptor agonists. Priming causes the rapid mobilisation of intracellular stores of receptors, including integrins, to the plasma membrane to facilitate migration from peripheral blood towards the site of infection. Subsequent activation at the site of infection, by engagement with complement proteins and/or immunoglobulins on the surface of opsonised bacteria, initiates phagocytosis and bacterial killing. This is normally followed by apoptosis of the neutrophil itself, which is then removed by resident tissue macrophages[2]. However, in inflammatory diseases such as rheumatoid arthritis (RA) neutrophils can be inappropriately primed and migrate towards sites of chronic infection, such as synovial joints, where they become activated by immune complexes, releasing ROS and proteases such as elastase directly onto host tissue leading to inflammation and damage to joints[2–4]. Production of neutrophil extracellular traps (NETs) in RA and other auto-immune conditions such as systemic lupus erythematosus (SLE), may lead to exposure of intracellular antigens such as dsDNA and citrullinated peptides, contributing to the development of auto-antibodies[3, 4]. NETs are also implicated in the inflammatory process, contributing to increased interferon-α production by plasmacytoid dendritic cells (pDCs) in SLE[5].

A recent resurgence in interest in metabolism in the context of immunity and inflammation (immunometabolism) has led to an increased understanding of the complex changes in metabolic regulation that take place in cells of the immune system during activation[6]. Changes in cellular activation, such as migration from the bloodstream to sites of inflammation or differentiation into tissue resident immune cells, can cause dramatic changes in nutrient and oxygen availability and place high metabolic demands upon immune cells[6, 7]. For example, it has been shown that M1 macrophages use glycolysis as their main source of energy, halting the TCA cycle and oxidative phosphorylation during differentiation from monocytes, whereas M2 macrophages display low glycolytic activity, rather relying on fatty acid oxidation to fuel the TCA cycle[7]. Fine-tuning of regulation of metabolic pathways during an inflammatory response is key for the generation of small molecule metabolites such as ATP, NADPH, nucleotides and amino acids, which are required rapidly and in high abundance during cellular activation[6, 7]. Dysregulation of metabolic control has been identified in inflammatory diseases such as rheumatoid arthritis[8], where changes in T cell glycolytic activity drives differentiation, hyperproliferation and hypermigration of T cell subsets[8–10].

Currently, there are the two major platforms for metabolomics analysis: Nuclear Magnetic Resonance (NMR) spectroscopy and Mass Spectrometry (MS)[11–13]. Whereas MS offers the advantage of high sensitivity, the approach is often targeted and only identifies specific metabolites of interest. In addition, MS requires the application of different chromatography techniques if one wishes to identify different classes of molecules. NMR metabolomics, on the other hand, offers a non-biased, non-separating approach to metabolic profiling which is faster and more cost effective, and which does not lead to sample loss[14–16]. Indeed, samples can be stored and re-run at a later date with a high level of reproducibility[17]. In addition, sample preparation for NMR metabolomics is less technically challenging, without the need for columns or complex optimisation[11], and reproducibility between different instruments is very good[18]. To date, NMR metabolomics protocols for biofluids[19, 20] and cell pellets[21] have been described.

The study of human neutrophils *in vitro* presents a number of technical challenges meaning that standard protocols for most assays must be optimised. Neutrophils are considerably more sensitive to manipulation than other cell types, and excessive centrifugation, agitation or rough handling of neutrophils during isolation from whole blood can lead to unwanted activation, and importantly, preparation protocols must take into account final cell purity, which should be free of contaminating PBMCs[22, 23]. Neutrophils must be isolated from whole blood within 1h of venepuncture to preserve the functional integrity of the cells and prevent cell death. Neutrophils are terminally differentiated cells and as such are short-lived, particularly in culture, with a life-span of less than 24h *in vitro*[2]. Neutrophils have lower levels of transcriptional activity compared to other leukocytes, and consequently, gene expression studies often have to use numbers of neutrophils at least ten-fold greater than other primary human cells or cell lines[22]. The level of metabolic activity in neutrophils compared to other primary human cells is unknown, but presumed lower, particularly in healthy, non-activated neutrophils. Intracellular reactions associated with neutrophil priming and activation occur in a matter of seconds[24], and so analysis of samples taken after suitable experimental time points are an important consideration when planning metabolomics experiments to detect neutrophil function. For example, one of the hallmarks of neutrophil function is the activation of the normally dormant NADPH oxidase during phagocytosis or other forms of activation, which can result in large changes in the NADP/NADPH ratio very rapidly after cell stimulation[1, 25]. This activation occurs rapidly, is sustained for several minutes and then declines. This is likely to be accompanied by transient and rapidly changing metabolic profiles.

The overall aim of this study was to optimise protocols for ^1^H NMR metabolomics to study changes in neutrophil metabolomics profiles during various forms of *in vivo* or *in vitro* activation. In particular, it was necessary to establish protocols to optimise sample preparation, metabolite extraction, and analysis that minimised the chemical and physical degradation of neutrophil metabolites and avoided the introduction of interfering compounds commonly used in isolation procedures. Importantly, we wanted to optimise our protocols for low amounts of starting material (<5 million neutrophils) to enable translation of the technology into clinical research, where ethical and practical restrictions often mean only small volumes of blood are available for analysis, particularly from paediatric samples. A recent application of NMR metabolomics to the study of neutrophils required at least 20 million cells per condition for metabolite extraction[26], which is in excess of the cell numbers routinely available from clinical studies. Our optimised protocol presented here has enabled, for the first time, metabolic profiling of less than 5 million neutrophils using NMR metabolomics. As there is no excessive preparation time required, this approach is capable of detecting the dynamic and transient formation of metabolites in neutrophils isolated from clinical samples.

## Methods

### Materials

HetaSep solution was from StemCell (Cambridge, UK); Ficoll-Paque was from GE Healthcare (Little Chalfont, UK); RPMI 1640 media was from Life Technologies (Paisley, UK); phorbol 12-myristate 13-acetate (PMA) and NADP was from Sigma (Gillingham, UK); HPLC grade acetonitrile (Thermofisher, UK), d4 trimethylsilyl propionate (TSP), and NaN_3_ (Sigma, Gillingham, UK).

### Neutrophil isolation

This study was carried out in accordance with the recommendations of the University of Liverpool Committee on Research Ethics. The protocol was approved by the University of Liverpool Committee on Research Ethics. All healthy subjects (4 males, 1 female, age range 25–57) gave written informed consent in accordance with the Declaration of Helsinki. Whole blood was collected into lithium-heparin vacutainers; the use of EDTA as an anticoagulant was avoided because extra resonance signals are observed in the NMR spectrum via the formation of complexes between EDTA and Ca^2+^ and Mg^2+^ present in plasma[13, 27]. Neutrophils were isolated within 15 min of blood collection. Whole blood was mixed with HetaSep solution at a ratio of 1:5 (HetaSep:whole blood) and incubated at 37°C for 30 min until the plasma/erythrocyte interphase was at approximately 50% of the total volume. The erythrocyte-free phase containing all nucleated blood cells was collected, and carefully layered on top of Ficoll-Paque solution at a ratio of 1:1, and then centrifuged at 500 g for 30 min. The peripheral blood mononuclear cells (PBMC) layer, plasma, and Ficoll-Paque solution, were carefully removed, leaving a neutrophil pellet (purity typically >97%)[22, 28–30]. The pellet was resuspended in RPMI-1640 media with 25 mM phosphate buffer pH 7.4. Remaining erythrocytes were removed by hypotonic lysis with ammonium chloride buffer for 3 min, and then centrifuged at 400 g for 3 min. The supernatant was removed, and neutrophils were re-suspended in RPMI-1640 with 25 mM phosphate buffer pH 7.4. Neutrophils were activated by the addition of phorbol 12-myristate 13-acetate (PMA, 0.1 μg/mL).

### Sample preparation for intracellular metabolite extraction

Our modified protocol to isolate neutrophils for metabolite extraction, was determined after a series of pilot studies. Steps that were deemed important for optimisation for the metabolomics pipeline, were briefly as follows (see Results section for detailed analysis): (1) To optimise the neutrophil cell number and the number of scans (NS), three different total cell numbers of neutrophils (2.5, 3.6 and 9.7 x 10^6^ cells per sample), were analysed using an increasing number of scans (NS) 128, 256, 512, 1024 and 2048. Analysis of technical triplicate samples was then performed to determine the reproducibility of spectral data in each experiment. (2) To optimise quenching of neutrophil metabolism by heating neutrophils prior to the snap freezing, 10^7^ healthy neutrophils were collected either with or without a heating step prior to the collection of cell pellet. The heating step was exposure of cell pellet to 100 ^o^C for 1 min, immediately before snap-freezing. (3) To determine any loss of NADP during sample preparation for NMR, purified NADP was added into the samples under three different conditions. First, 10 μg NADP (in 50 μL PBS) was added into the cell pellets of 9.7 x 10^6^ neutrophils before the heating steps. The cell pellets then went through the extraction and lyophilisation procedures. Second, 10 μg NADP was added to 50 μL PBS (no cell pellets). The NADP-spiked samples were then heat shocked, and extracted and lyophilised. Third, 20 μg NADP (in 200 μL PBS), without any processing, was analysed as a positive control.

To prepare cell pellets for metabolite extraction, neutrophils were centrifuged at 1000 g at 25 ^o^C for 2 min. The supernatant was aspirated, and cell pellets were resuspended with ice-cold PBS, then centrifuged at 1000 g at 25 ^o^C for 2 min. The supernatant was discarded, while the pellets were heated at 100 ^o^C for 1 min, and then were snap-frozen in liquid nitrogen. All samples were stored at -80 ^o^C prior to intracellular metabolite extraction.

### Intracellular metabolite extraction

Cell samples were extracted by addition of 50:50 v/v ice cold HPLC grade acetonitrile:water at 500 μL per cell pellet (a constant volume irrespective of pellet size), followed by a 10 min incubation on ice. Then samples were sonicated three times for 30 s at 23 kHz and 10 μm amplitude using an exponential probe, with 30 s rest between sonications in an ice water bath. Sonicated samples were centrifuged at 12000 g for 5 min at 4°C and the supernatant transferred to cryovials, flash frozen in liquid N_2_ and lyophilised[13].

### Sample preparation for NMR

Each lyophilised sample was resuspended in 200 μl of 100 mM deuterated sodium phosphate buffer pH 7.4, with 100 μM d4 trimethylsilyl propionate (TSP) and 0.05% NaN_3_. Each sample was vortexed for 20 s and centrifuged at 12000 g for 1 min at 20°C. 180 μl of each cell extract sample was transferred to 3 mm (outer diameter) NMR tubes for acquisition.

### NMR acquisition and spectral processing

The samples were acquired using a 700 MHz NMR Avance IIIHD Bruker NMR spectrometer equipped with a TCI cryoprobe. Samples were referenced to trimethylsilylpropanoic acid (TSP) at 0 ppm. Chenomx, Bruker TopSpin and AMIX software were used to identify metabolites and process spectra. Samples were analysed using the 1D Carr-Purcell-Meiboom-Gill (CPMG) edited pulse sequence technique (to selectively detect low molecular weight metabolites, less than 1,000 Da in the acquired spectra). Different total cell numbers of neutrophils (2.5, 3.6 and 9.7 x 10^6^ cells per sample), were analysed decreasing signal-to-noise while increasing experiment time using an increasing number of scans (NS), i.e. 128, 256, 512, 1024 and 2048, requiring 15 min, 30 min, 1h, 2h and 4 h acquisition time per sample, respectively. Temperature was calibrated to 25 ^o^C (with margin of error of 0.1 ^o^C) *via* a methanol thermometer[31] prior to each day of acquisition; receiver gain was kept constant for all acquisitions. Spectra were automatically phased, referenced and baseline corrected in Topspin 3.1 *via* apk0.noe routine (Bruker, UK). Metabolomics data have been deposited to the EMBL-EBI MetaboLights database[32] with the identifier MTBLS658. The complete dataset can be accessed here: https://www.ebi.ac.uk/metabolights/MTBLS658.

### Spectra processing and quality control

The spectra were assessed to conform to minimum quality criteria as outlined by the Metabolomics Society[33] to ensure consistent linewidths, baseline corrections and water suppression. All spectra passing quality criteria were then divided into ‘buckets’ or 'bins' that were defined globally by the peak limits using Amix® software. All peaks, both annotated in Chenomx (*via* manual analyses in TopSpin and Chenomx software) and unknown, were included in the bucket table (see example spectra in S1 Fig and metabolite annotations in S1 Table).

### Metabolite annotation and identification

Metabolites in the ^1^H NMR spectra were initially tentatively annotated using the metabolite discovery software Chenomx (Chenomx, Canada) and, where possible, their identities confirmed using an in-house library of metabolite spectra[34]. Annotation level is indicated in the Metabolights deposition.

### Metabolomics statistical analysis

Statistical analyses were performed using the online programme, Metaboanalyst® 3.0 (http://www.metaboanalyst.ca/)[35, 36], which uses the R package of statistical computing software. The bucketed experimental data were normalised by the total spectral intensity and additionally Pareto scaled (for multivariate analysis). NMR data were found to show a normal distribution and therefore no data transformation was required. Univariate analysis was by ANOVA with application of a False-Discovery Rate (FDR) adjusted p-value of 0.05. Multivariate analysis was *via* unsupervised Principal Component Analysis (PCA) followed by Partial Least Squares Discriminant Analysis (PLS-DA) validated *via* leave one out cross-validation to automatically determine optimal number of components as well as the quality of the model (described in terms of accuracy, R2 and Q2). Hierarchical cluster analysis was carried out using MeV[37] using Euclidean clustering and average linkage.

### Metabolomics pathway analysis

Pathway analysis was carried out using the list of significantly different metabolites between PMA-treated and untreated neutrophils using Metaboanalyst® and the methodology of Metabolite Set Enrichment Analysis[36, 38], with reference to the KEGG (Kyoto Encyclopedia of Genes and Genomes) metabolic pathway database (http://www.kegg.jp/kegg/pathway.html) for human metabolic pathways[36] and current understanding of biochemical pathways[39]. Pathway enrichment was determined by Hypergeometric test and reported with an FDR-adjusted p-value.

## Results

### Optimising protocols for NMR metabolomics analyses of human neutrophils

No established protocols have yet been developed for the measurement of the metabolomic profiles of <5 million human neutrophils using NMR. Our first objective was to optimise these procedures in view of both the challenges and the potential advantages of different protocols, and of the difficulties in working with human neutrophils at the low cell numbers that may be obtained from clinical samples. Also, in light of the very rapid and transient nature of changes in neutrophil metabolism following stimulation, we wanted to develop a rapid and efficient extraction methodology with minimal perturbation.

### Minimising processing time and wash steps

**Fig 1:**
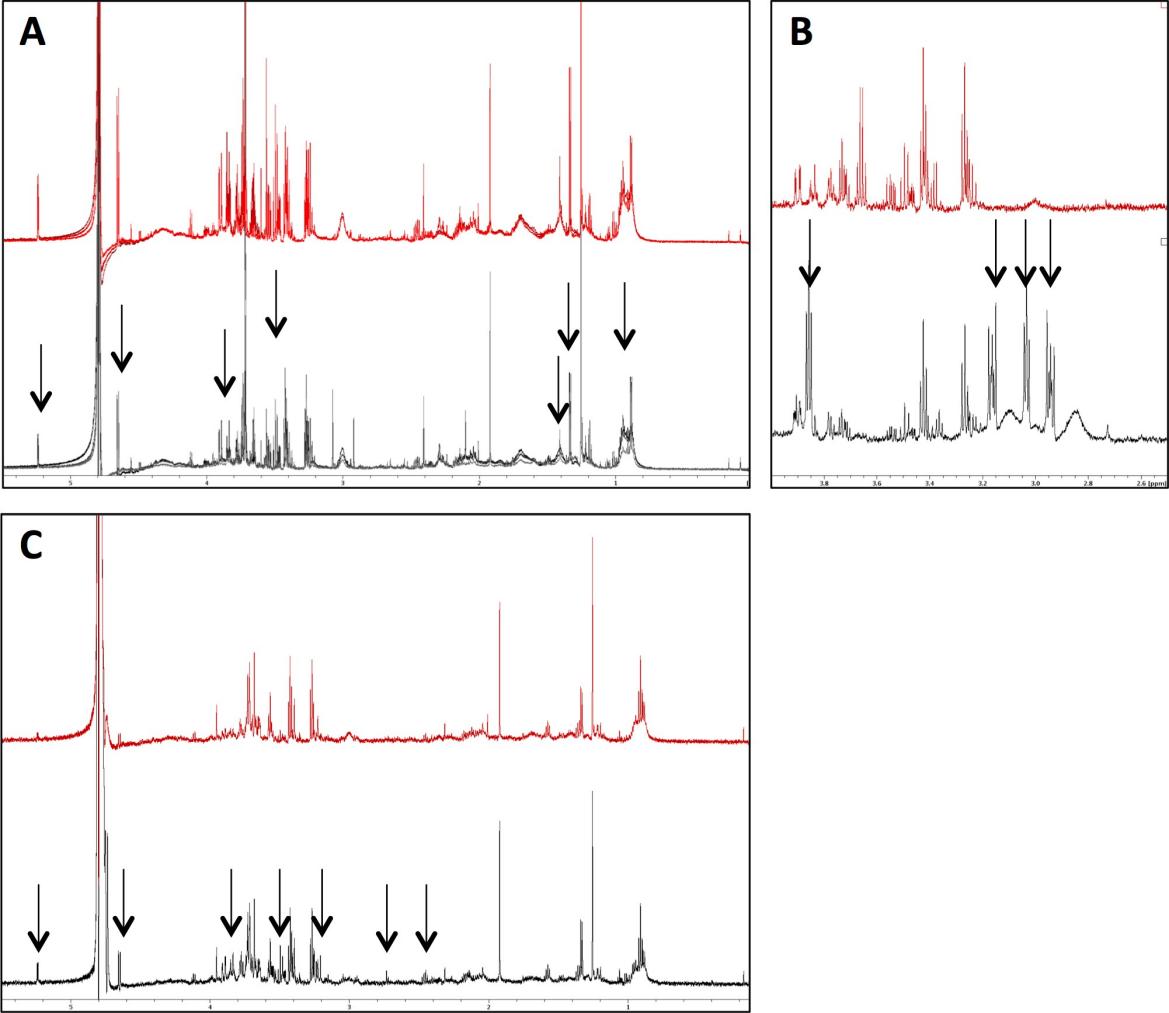
Optimisation of neutrophil isolation protocols for NMR metabolomics. (A) Spectra (n = 3) shown for neutrophil isolation protocols including (grey spectra) or omitting (red spectra) a wash step immediately after Ficoll-Paque separation. Metabolite loss in the washed samples is indicated by arrows. (B) Spectra (n = 3) show neutrophils incubated with media containing phosphate buffer (red spectra) or HEPES buffer (grey spectra). Multiple contaminating peaks from HEPES are indicated by arrows. (C) Spectra (n = 3) are shown for sample preparation including (grey spectra) or excluding (red spectra) a heat-shock step immediately prior to snap freezing of pellet. Metabolite degradation/turnover was prevented by heat shock as indicated by arrows.

During the isolation process, all washing steps were minimised as much as appropriate without compromising the numbers of cells collected or carry over of media. For example, the washing step after centrifugation in Ficoll-Paque reagent was eliminated. In order to investigate the effect of washing steps, the neutrophil suspensions were equally distributed into two tubes, at a concentration of 3.5x10^6^ cells/mL in 3mL of RPMI-1640 media. Both tubes were centrifuged at 500 g for 3 min. In one tube (washed sample), the supernatant was discarded and 3 mL RPMI-1640 media was added. After the red blood cell lysis step, the cell pellet was resuspended and recounted. The cell concentration had decreased to 3x10^6^ cells/mL. In another tube (non-washed sample), the supernatant was not discarded but was used again to resuspend the cell pellet. The cell suspensions were then aliquoted into three tubes for technical replicates. The NMR spectra were visibly different, and metabolite loss was clearly evident in the washed sample (Fig 1A). Variation between technical replicates was also visibly greater in samples including a wash step, indicating loss of cells and/or metabolites during washing. Direct observation of contributing peaks from contaminating Ficoll-Paque was not present in the spectra (S1 Fig).

### Use of HEPES as media buffer

NMR metabolomics has the potential to detect intracellular metabolites at very low levels, for example as low as 500 ng of pure compound[40]. However, these low quantity metabolites are detected as low intensity peaks, which can be easily obscured by the presence of high concentration metabolites, some of which may commonly be contaminants. As the NMR technique is a very sensitive tool, all possible contaminants, including chemicals and disinfectants used in cell preparation/isolation, were considered and then eliminated from the isolation/extraction protocol, wherever possible. RPMI media with HEPES buffer is routinely used in our normal protocols for the preparation of human neutrophils, in order to maintain the pH of the media around 7.2–7.5, and maintain viable, unactivated human neutrophils[41, 42]. Moreover, altered pH of the samples could influence the chemical shifts of the NMR spectrum[43]. However, residual HEPES in the neutrophil extracts (from the RPMI medium) is undesirable, due to the buffer exhibiting multiple high peaks, in the ^1^H NMR spectra (Fig 1B). Therefore, in all subsequent experiments, 25 mM phosphate buffer was added into the media in place of HEPES, to maintain the pH at 7.4. Phosphate buffer does not give rise to ^1^H NMR signals[13], thus not impacting the overall NMR spectrum, and as such phosphate buffer was used in all subsequent neutrophil NMR metabolomics experiments.

### Heat quenching of metabolites prior to extraction

We next determined the effect of quenching neutrophil metabolism by heating neutrophils prior to snap freezing. In these experiments, neutrophils were heated to 100 ^o^C for 1 min in order to denature metabolic enzymes (which could be released during extraction) and hence stabilise all metabolites, protecting them from further cellular reactions. Neutrophils were incubated with the protein kinase C activator phorbyl 12-myristate 13-acetate (PMA, 0.1 μg/mL) for 10 min to stimulate metabolic activity and production of ROS, after which heat shock was applied to one sample while another sample was extracted without the heat shock treatment. Heat shock treated samples demonstrated overall higher levels of metabolites than spectra obtained from the samples extracted without the heat shock step (Fig 1C).

### Cell number and number of scans

Finally, optimisation of neutrophil cell number was necessary, as the total numbers of neutrophils that can be routinely obtained from the blood or biofluids of patients and healthy donors can be low. For clinical samples, particularly from paediatric patients, the total number of neutrophils obtained from a single blood sample may be as low as 2x10^6^ cells. Therefore, it was critical to determine the minimum number of neutrophils required to detect the highest possible number of metabolites in the NMR spectra and to generate optimal signal-to-noise ratio.

**Fig 2:**
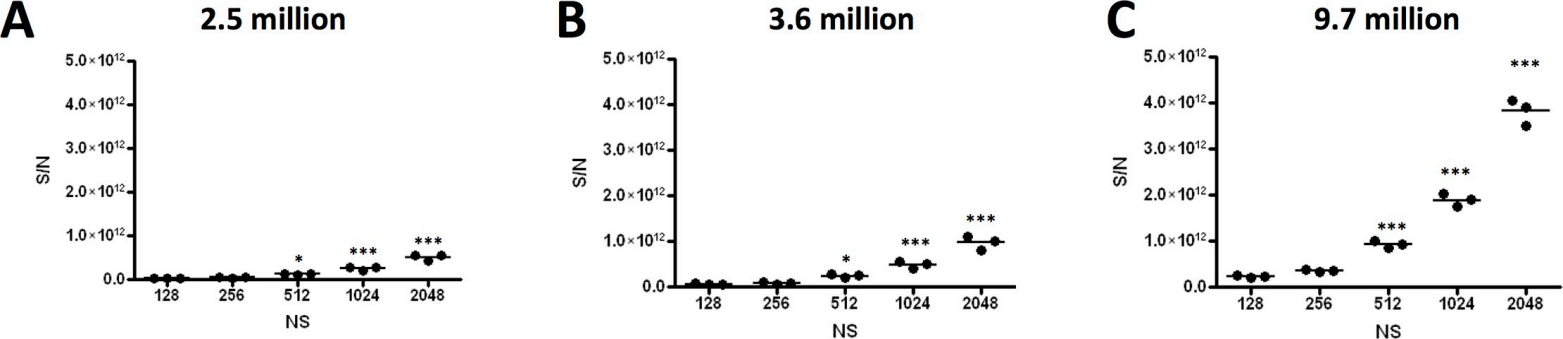
Optimisation of cell number and number of scans required for neutrophil metabolomics. Principle Component Analysis was carried out on triplicate samples of different neutrophil cell concentrations (A) 2.5x10^6^, (B) 3.6x10^6^ and (C) 9.7 x 10^6^ cells. Each sample was measured at an increasing number of scans: 128, 256, 512, 1024 and 2048. Significant increase in signal to noise (S/N) was observed at 512NS (ANOVA with Tukey’s post-hoc test, n = 3, *p<0.05, ***p<0.001).

Neutrophils were prepared in technical triplicates at three different cell numbers (2.5, 3.6 and 9.7x10^6^ cells) and analysed using five different numbers of scans (NS) (128, 256, 512, 1024 and 2048). The non-destructive nature of NMR metabolomics meant that we were able to run the same samples at five different NS for this optimisation step. Analysis of the signal to noise (S/N) ratio showed a significant increase in S/N with the increasing NS. This became significant for all cell concentrations at 512 NS (Fig 2A p<0.0001 ANOVA, post-hoc *p<0.05, **p<0.01, ***p<0.001).

### Detection of low-level metabolites

**Fig 3:**
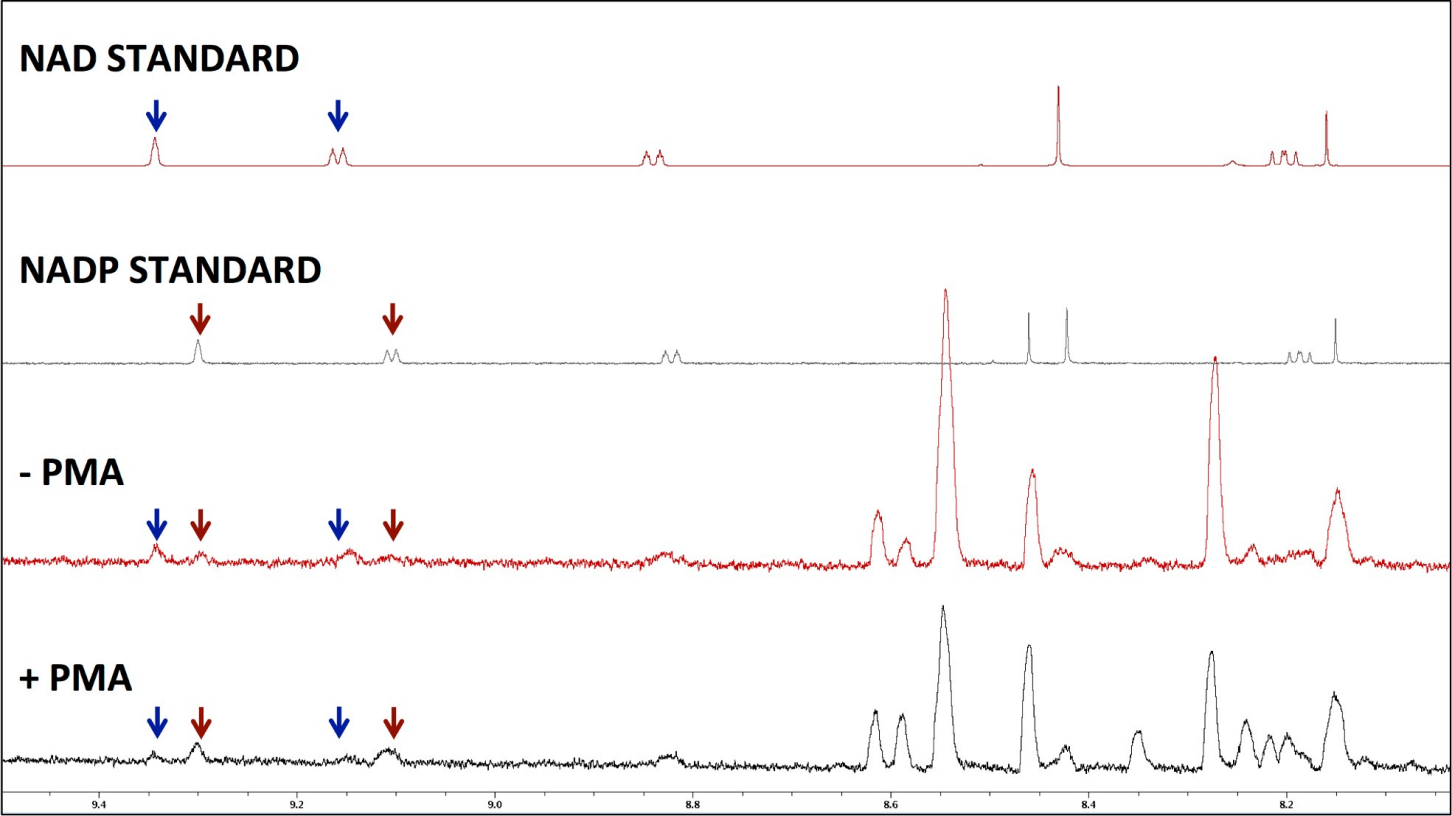
Detection of NAD and NADP^+^ in neutrophil spectra. Neutrophils were incubated in the absence or presence of PMA (0.1 μg/mL) for 15 min. An increase in the ratio of NADP^+^ to NAD was observed after addition of PMA. Spectral peaks for NAD (blue arrow) and NADP^+^ (red arrow) are shown in both neutrophil preparations (+/- PMA) and cell-free standards.

Neutrophils possess sophisticated mechanisms for the phagocytosis and killing of bacteria. These include the generation of ROS *via* the NADPH oxidase, which is rapidly activated during cell stimulation[1]. In order to fuel the NADPH oxidase, dynamic and rapid changes in the concentrations and ratios of NAD, NADP and NADPH take place during neutrophil activation. In our preliminary experiments (at 256 NS), the spectral peaks representing NAD and NADP were barely detectable (S2 Fig). Therefore, experiments with increased signal-to-noise were analysed to determine whether the detection of the NAD spectral peaks could be enhanced. The study revealed the presence of more prominent NAD and NADP spectral peaks at 4096 scans for minimal number of cells (2.5 x 10^6^ cells). Using this approach, NAD and NADP were detected, but still at low levels. We found that by increasing the cell concentration to ≥3.6 million cells and increasing the number of scans to 512, not only could we detect NAD and NADP in neutrophil extracts, but importantly a change in the ratio of NAD to NADP was clearly observed when samples were treated with PMA (Fig 3).

### NMR Metabolomics analysis of healthy human neutrophils

#### Metabolomic analysis of unactivated and activated healthy human neutrophils

**Fig 4:**
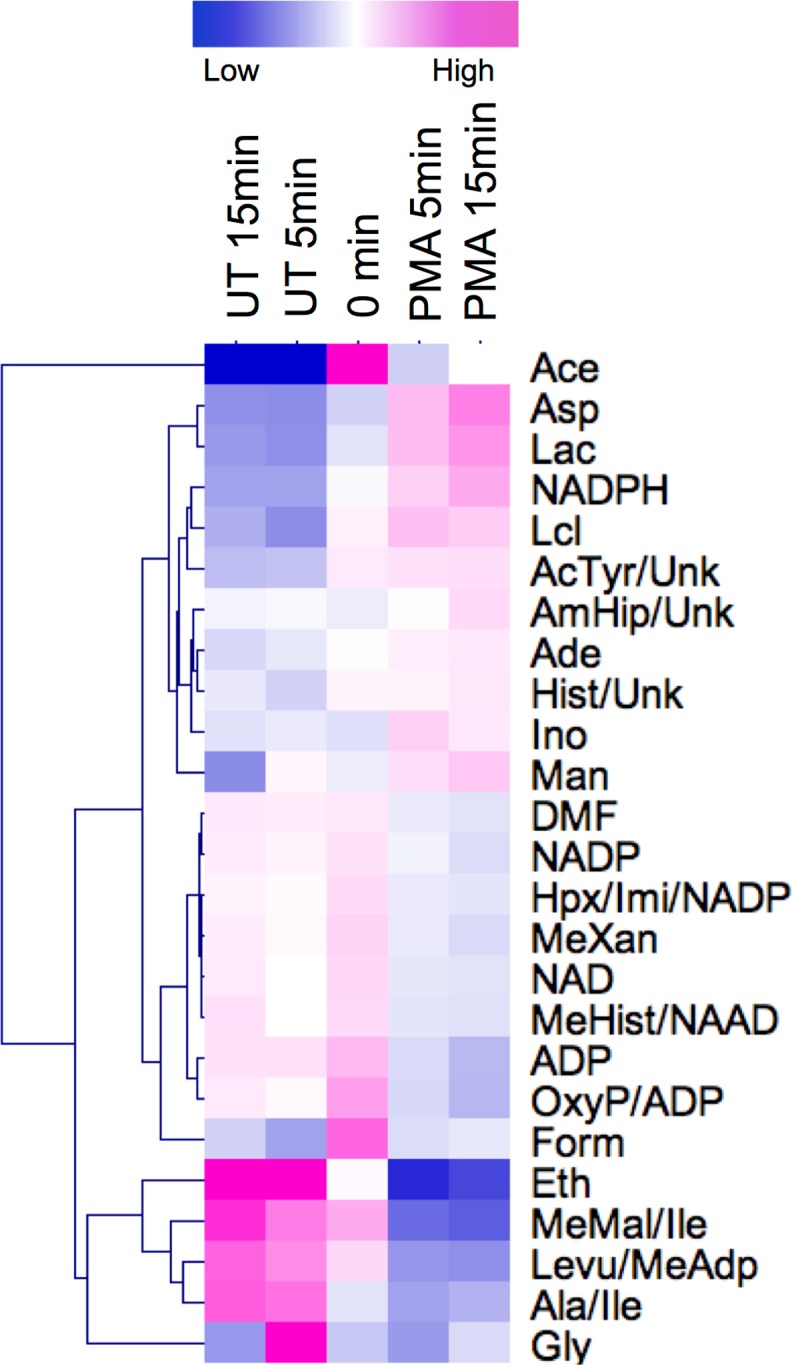
Heatmap of metabolites with at least 1.5-fold change (increase or decrease) from 0h following treatment with or without PMA for 5 and 15 min. Ace = acetate, AcTyr = acetyltyrosine, Ade = adenine, ADP = adenosine diphosphate, Ala = alanine, AmHip = aminohippurate, Asp = aspartate, Ery = erythritol, Eth = ethanol, DMF = dimethylformamide, Form = Formate, Gly = glycine, Hist = histamine, Hpx = hypoxanthine, Ile = isoleucine, Imi = imidazole, Ino = inosine, Lac = lactate, Lcl = lactulose, Levu = levulinate, Man = mannose, MeAdp = methyladipate, MeHist = methylhistidine, MeMal = methylmalonate, MeXan = methylxanthine, NAAD = nicotinic acid adenine nucleotide, NAD = nicotinamide adenine dinucleotide, NADP = nicotinamide adenine dinucleotide phosphate, NADPH = nicotinamide adenine dinucleotide (reduced), OxyP = oxypurinol, Unk = unknown. Mean fold change shown for n = 2 or 3 biological replicates measured in technical triplicates.

In order to determine the metabolite profile of unactivated, healthy neutrophils and investigate the metabolic changes that take place when neutrophils are activated, we stimulated healthy neutrophils with PMA (0.1 μg/mL) for 5 and 15 min. Metabolites were extracted from unactivated and PMA-activated neutrophils as per our optimised protocol described above. The NMR spectra were divided into 327 spectral bins (based on representative spectra from samples +/- PMA treatment), of which 287 (87.7%) were assigned to 110 metabolites including: amino acids, peptides and analogues; carbohydrates, carbonyls and alcohols; nucleotides, nucleosides and analogues; lipids and lipid-like molecules; benzenoids; and other organic compounds (Table 1). Univariate analysis of 5 min and 15 min timepoints ± PMA identified 12 spectral buckets that were significantly increased or decreased after PMA treatment (FDR<0.05, Table 2). The metabolites represented by the significantly changing spectral buckets were ADP and aspartate and/or homocysteine as well as some unidentified metabolites. Fifty-four spectral buckets had a fold-change in abundance (increase or decrease) of at least 1.5 fold at 5 or 15 min post PMA treatment, including NAD, NADP and NADPH. The levels of NAD and NADP decreased by -1.8 and -1.6 fold respectively, whereas NADPH increased by 1.7 fold with 15 min PMA treatment. Several of these buckets related to multiple spectral peaks from the same metabolite, for example NAD was represented by four different buckets, and some single buckets were assigned to multiple metabolites, for example alanine and isoleucine (Fig 4, S3 Fig, S1 Table). In addition, the levels of many metabolites changed over 15 min in untreated neutrophils, including aspartate, lactate and NADPH which decreased, and NAD and NADP which increased (Fig 4).

**Table 1:** List of 110 metabolites detected in healthy neutrophils by ^1^H NMR spectroscopy (n = 5 biological replicates).

*Amino acids*, *peptides*, *and analogues* 1-Methylhistidine, 3-Methylhistidine, Acetylglycine, Betaine, Dimethylglycine, gamma-Glutamylphenylalanine, Glutathione, Glycine, L-Alanine, L-Aspartic acid, L-Glutamic acid, L-Histidine, Homocysteine, Isovalerylglycine, L-Isoleucine, L-Phenylalanine, L-Tyrosine, L-Valine, N-Acetyl-L-aspartic acid, N-Acetyl-L-tyrosine, N-Alpha-acetyllysine, N-Phenylacetylphenylalanine, Phenylacetylglycine, Saccharopine
*Carbohydrates*, *carbonyls and alcohols* Beta-N-Acetylglucosamine, Dihydroxyacetone, D-Glucose, D-Glucuronic acid, D-Mannose, Erythritol, Ethanol, Lactulose, L-3-Hydroxykynurenine, L-Kynurenine, Methanol
*Nucleosides*, *nucleotides*, *and analogues* ADP, AMP, ATP, Cytidine, dADP, dIDP, Guanosine, Guanosine triphosphate, Inosine, NAD, NADH, NADP, NADPH, Nicotinic acid adenine dinucleotide, S-Adenosylhomocysteine, Uridine diphosphate-N-acetylglucosamine, Uridine diphosphate glucose, Uridine diphosphate glucuronic acid, Uridine 5'-diphosphate, Uridine 5'-monophosphate
*Lipids and lipid-like molecules* 2-Hydroxy-3-methylbutyric acid, 2-Hydroxyvaleric acid, 3-Hydroxyisovaleric acid, 3-Hydroxymethylglutaric acid, 3-Methyladipic acid, Methylsuccinic acid, Monomethyl glutaric acid, Thymol
*Benzenoids (benzene derivatives and phenols)* 3-Hydroxymandelic acid, 3-Hydroxyphenylacetic acid, 4-Aminohippuric acid, Acetaminophen, Homovanillic acid, o-Cresol, Ortho-Hydroxyphenylacetic acid, p-Cresol, Phenylacetic acid, p-Hydroxyphenylacetic acid, Salicylic acid, Syringic acid, Tyramine, Vanillylmandelic acid
*Other organic compounds* 1,3-Dimethyluric acid, 2-Ketohexanoic acid, 3-Methylxanthine,5-Hydroxyindoleacetic acid, 5-Hydroxy-L-tryptophan, Acetamide, Acetic acid, Adenine, Anserine, Carnosine, Choline, Desaminotyrosine, Formic acid, Histamine, Hypoxanthine, Indoxyl sulfate, Imidazole, Indoleacetic acid, Indolelactic acid, Isocitric acid, L-Carnitine, L-Lactic acid, Levulinic acid, Methylmalonic acid, Niacinamide, N,N-Dimethylformamide, Oxypurinol, Phenyllactic acid, Phosphorylcholine, Pyridoxine, Riboflavin, Taurine, Xanthine

**Table 2:** Univariate analysis of significantly altered metabolite changes after 5 or 15 min PMA treatment (FDR < 0.05, n = 3 biological replicates measured in technical triplicates).

	PMA 5 min	PMA 15 min	p-value (-log10)	FDR
Unknown	+	+	9.6783	6.8x10^-8^
ADP	+	+	6.0101	1.6x10^-4^
Oxypurinol/ADP	+	+	5.7127	2.1x10^-4^
ADP or ATP	+	+	5.4374	2.9x10^-4^
Unknown	+	+	5.1849	4.6x10^-4^
Homocysteine/Aspartate	+	+	4.8621	7.5x10^-4^
Homocysteine/Aspartate/Anserine	+		4.5884	1.2x10^-3^
ATP/Unknown	+	+	4.537	1.2x10^-3^
Aspartate	+	+	4.4519	1.3x10^-3^
Aspartate	+	+	3.3857	1.3x10^-2^
Unknown	+	+	2.851	4.2x10^-2^
N-acetylaspartate/homocysteine/unknown	+	+	2.7527	4.8x10^-2^

+ = metabolite significantly altered

#### Multivariate analysis of activated neutrophils

**Fig 5:**
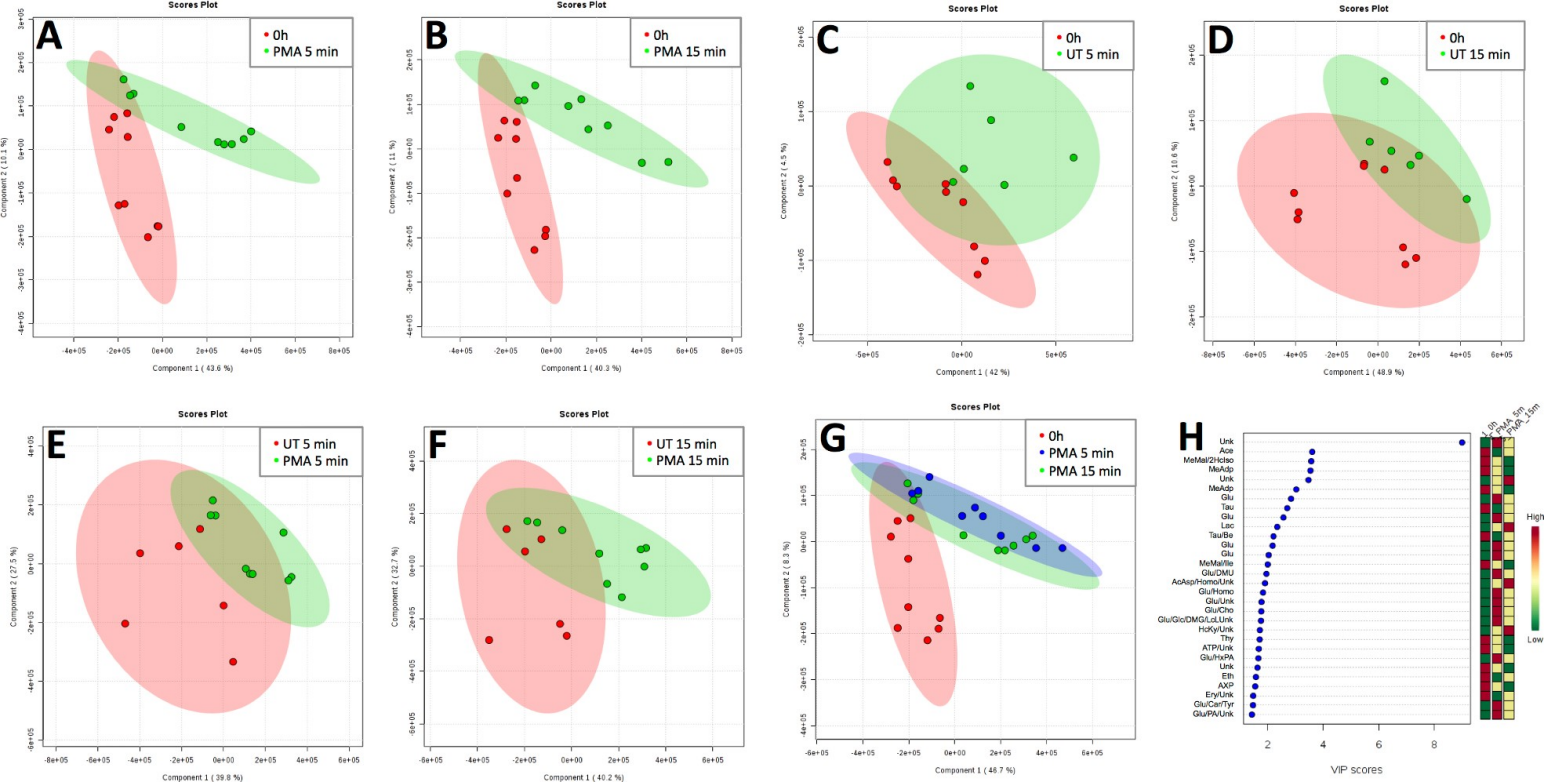
Change in neutrophil metabolome induced by PMA. Neutrophils were incubated in the presence (A,B,E,F,G) or absence (C,D,E,F) of PMA (0.1 μg/mL) for 5 (A,C,E,G) and 15 min (B,D,F,G) (n = 2 or 3 biological replicates measured in technical triplicates). Supervised multivariate analysis by PLS-DA segregated PMA-treated samples. Shading represents 95% confidence region. Scores plot is shown for components 1 and 2. (H) Variable Importance in Projection (VIP) analysis suggested influential metabolites for this separation are increasing levels of lactate, glucose and 3-hydroxykyneurinine, and decreasing levels of acetate, methylmalonate, 3-methyladipate and taurine.

Multivariate analysis (PLS-DA, partial least square discriminant analysis, 5 components) showed that when neutrophils were treated with PMA, the metabolic profile changed significantly, as evidenced by the quality of discrimination between 0 and 5 min (Fig 5A, Q2 0.7932, R2 0.9685, accuracy 0.9444) and 0 and 15 min (Fig 5B, Q2 0.93712, R2 0.98421, accuracy 1.0) whereas differences between 5 and 15 min of PMA treatment were less distinct (S4 Fig, Q2 0.39248, R2 0.91143, accuracy 0.83333). It should be noted that the metabolome of untreated neutrophils changes over time, as can be seen from the analysis of untreated cells at 0 and 5 min (Fig 5D, Q2 0.32754, R2 0.8382, accuracy 0.8) or 0 and 15 min (Fig 5E, Q2 0.66767, R2 0.9411, accuracy 0.86667); however the model for 5 and 15 minutes for untreated cells did not distinguish the two groups (S5 Fig, Q2–6.4346, R2 0.93566, accuracy 0.16667). Discriminant analysis of PMA treated neutrophils versus untreated neutrophils modelled differences at 5 min (Fig 5G, Q2 0.64496, R2 0.92841, accuracy 0.93333) and 15 min (Fig 5H, Q2 0.64488, R2 0.98587, accuracy 0.93333). Comparison of 0 min vs PMA at 5 min and PMA at 15 min also clearly indicates the time-dependent effect of PMA on metabolism (Fig 5I, Q2 0.79897, R2 0.93934, accuracy 0.66667) with Variable Influencing Projection (VIP) analysis indicating influential metabolites for this separation are increasing levels of lactate, glucose and 3-hydroxykyneurinine, and decreasing levels of acetate, methylmalonate, 3-methyladipate and taurine (Fig 5J). Of note, the levels of glucose rapidly increased from 0h to 5 min following PMA stimulation, and then fell again following 15 min PMA stimulation.

#### Biological context

**Fig 6:**
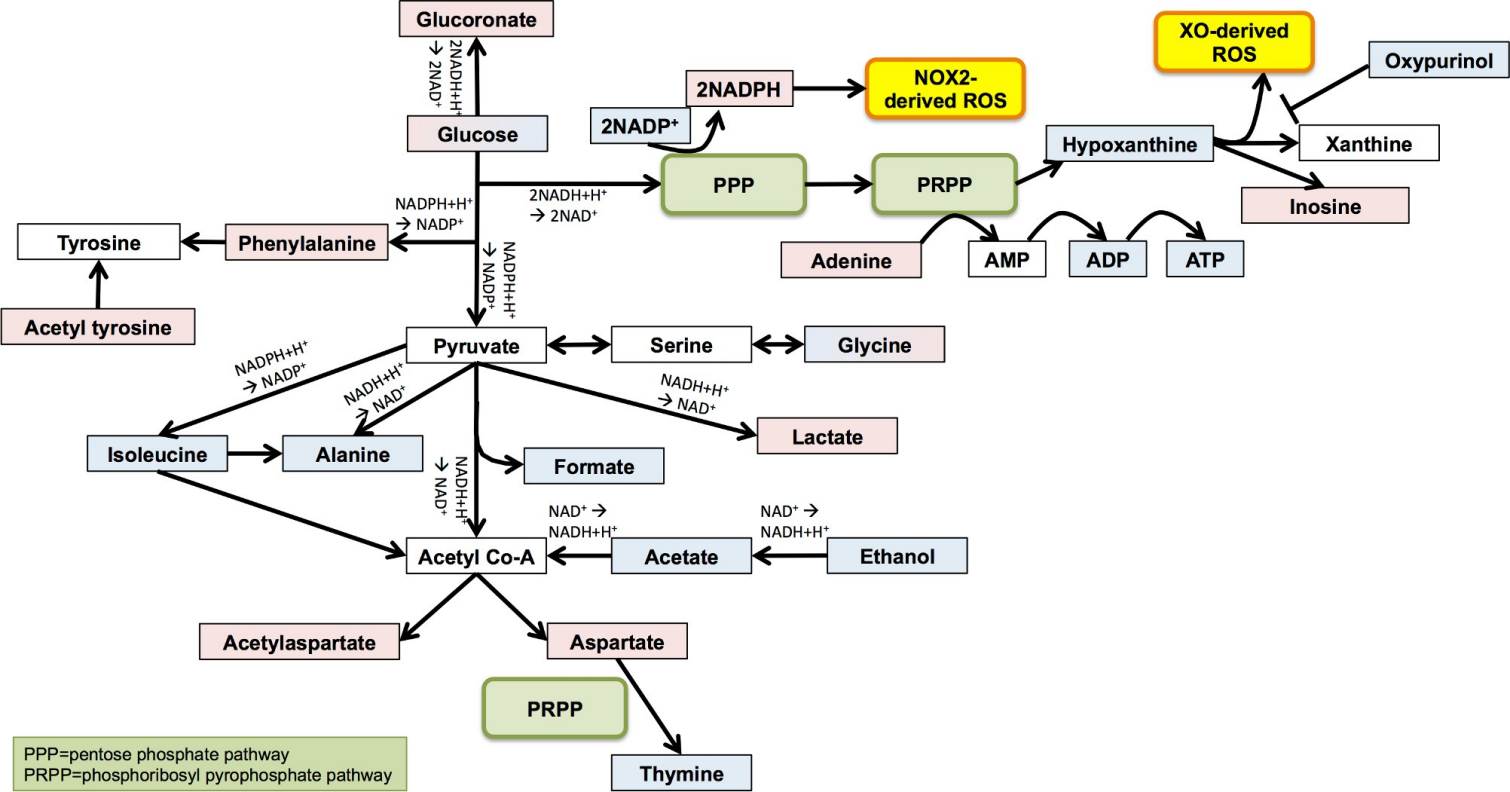
Pathway scheme depicting metabolite changes during neutrophil activation with PMA. Metabolites shown in blue decreased following PMA stimulation and metabolites shown in red increased; gradient red/blue shading represents an increase following 5 min and then a decrease by 15 min with PMA activation, and *vice versa*. Metabolites shown in white were not detected but are key components of the pathway.

Pathway analysis revealed that the most significant metabolomic changes in PMA-activated neutrophils involved nicotinate & nicotinamide metabolism, aminoacyl-tRNA biosynthesis and glycolysis (Table 3). We combined the results of the pathway analysis with our univariate and multivariate analyses into a single metabolic flow diagram (Fig 6) to enable interpretation of the interactions between the metabolites and the different metabolic pathways. Our analysis revealed a redirection of metabolic activity from glycolysis to the pentose phosphate pathway (PPP) and the phosphoribosyl pyrophosphate pathway (PRPP) when neutrophils were activated by PMA. Both the PPP and the PRPP fuel ROS production *via* the NADPH oxidase and xanthine oxidase respectively.

**Table 3:** Pathway analysis of metabolites changing 1.5 fold in the presence of PMA.

	Hits/Total	P value	FDR
Nicotinate & nicotinamide metabolism	4/44	2.13x10^-3^	0.092617
Aminoacyl-tRNA biosynthesis	5/75	2.32x10^-3^	0.092617
Glycolysis or Gluconeogenesis	3/31	6.81x10^-3^	0.18172
Nitrogen metabolism	3/39	1.29x10^-2^	0.25847
Taurine & hypotaurine metabolism	2/20	2.64x10^-2^	0.36115
Purine metabolism	4/92	2.85x10^-2^	0.36115
Selenoamino acid metabolism	2/22	3.16x10^-2^	0.36115
Alanine, aspartate & glutamate metabolism	2/24	3.72x10^-2^	0.37161
Phenylalanine, tyrosine & tryptophan biosynthesis	2/27	4.61x10^-2^	0.39444
beta-Alanine metabolism	2/28	4.93x10^-2^	0.39444

## Discussion

The aim of this research was to develop protocols for the study of human neutrophils using ^1^H NMR metabolomics. We decided to use the NMR methodology to study neutrophil metabolomics, as this technique, unlike mass spectrometry-based techniques, allows quantitative analyses of untargeted metabolomics profiles. NMR spectra of biofluids and pattern recognition methods in metabolomics has enabled the identification and quantitation of metabolites in different biological samples including biofluids such as urine[19], serum[20] and synovial fluid[44], and in cell pellets[21] including neutrophils[26], thus providing highly informative data about the functional state of living organisms, including humans.

A key aim of our study was to develop protocols to allow the measurement of metabolites in neutrophils isolated from low volumes of blood (<5 million neutrophils) to enable future analysis of clinical samples, which are often low in volume. As such our optimised protocol differs from that recently published which requires 20 million neutrophils for metabolite extraction[26]. We were able to prevent metabolite loss by decreasing the number of wash steps during the neutrophil isolation protocol and, importantly, inclusion of a heat shock step prior to metabolite extraction. Protocols were developed to take into account of the low levels of metabolites in human neutrophils, and to develop optimal conditions for the detection of metabolites such as NADP, which have high rates of turnover during activation of neutrophils. We also considered ways to minimise undesirable activation of neutrophils during the isolation protocol. In order to optimise protocols for neutrophil metabolomics, it was necessary to study both unactivated (freshly-isolated healthy control) and activated neutrophils.

It was decided to use PMA as the activating factor for *in vitro* optimisation experiments. PMA is an analogue of diacylglycerol, which rapidly activates protein kinase C (PKC), generating a respiratory burst through activation of the NADPH oxidase[45]. It is used experimentally to activate ROS production (*via* the respiratory burst) from primed and unprimed neutrophils, and is a potent activator of neutrophil extracellular trap formation (NETosis)[29, 46, 47]. The major changes in metabolism observed in PMA-treated neutrophils centred around the glycolysis pathway. A dynamic regulation of glucose levels in neutrophils in response to PMA was observed; after 5 min PMA treatment glucose levels increased compared to 0h neutrophils, and after 15 min glucose levels were lower compared to 5 min with PMA-stimulation. This likely represents a rapid import of glucose from culture media at 5 min (or else glycogenolysis[48]), and then rapid metabolism of glucose by 15 min *via* the pentose phosphate pathway (PPP) (also called the hexose monophosphate shunt). The PPP is a key pathway in the dehydrogenation of NADP to NADPH, with NADPH being the key electron donor for ROS production *via* the NADPH oxidase. We observed decreases in the abundance of downstream metabolites of glycolysis (e.g. isoleucine, alanine, formate, acetate and ethanol) following PMA stimulation, confirming the redirection of glucose metabolism to the PPP to fuel NADPH production for rapid activation of the NADPH oxidase leading to ROS production. Whilst pathway analysis predicted the decrease in these metabolites may be related to gluconeogenesis, is has previously been established that neutrophils are unable to produce glucose *via* gluconeogenesis[49], and that their glucose supply is provided solely *via* uptake from the blood or culture media or glycogenolysis[48]. The high levels of lactate observed in our study appear to support this theory, as lactate is the main substrate for gluconeogenesis[39]. A decrease in hypoxanthine may also indicate ROS production *via* xanthine oxidase (XO)[39] in response to PMA. Oxypurinol, an inhibitor of XO-derived ROS was also decreased by PMA-stimulation. Ambiguity in metabolite association with specific pathways was mitigated by metabolite set enrichment analysis. Nevertheless, incomplete databases[50] may lead to gaps in the association of metabolites with certain pathways, and it is clear that lactate, hypoxanthine and oxupurinol are associated with many metabolic pathways (Human Metabolome Database (HMDB)[51–54] accession numbers HMDB0000190, HMDB0000157 and HMDB0000786 respectively).

No metabolites relating to the TCA cycle were detected in this study. Unlike other leukocytes, neutrophils obtain all their energy *via* aerobic glycolysis[55–57] and the TCA cycle is entirely absent. Moreover, neutrophil mitochondria maintain transmembrane potential *via* the glycerol-3-phosphate shuttle rather than oxidative phosphorylation and lack respiratory chain super-complex organisation[57]. As a consequence, neutrophil mitochondria contribute little to ATP synthesis[48] and function predominantly in the regulation of apoptosis and migration[58, 59]. In the presence of glucose, ATP is generated from lactate produced during glycolysis. The disappearance of ATP during neutrophil functions such as phagocytosis is irreversible, with no detectable increase in ADP or AMP[48]. PMA has previously been shown to increase lactate production[46] in line with the findings of our study. Metabolic reprogramming of neutrophils to aerobic glycolysis during maturation enables these cells to survive at sites of inflammation, which typically have low oxygen tensions, preventing synthesis of ATP *via* oxidative phosphorylation[56].

PMA is a well-described agonist of neutrophil extracellular trap (NET) production *in vitro*[46, 47]. NET production in response to PMA has been demonstrated to be dependent upon glucose and glycolysis; in the absence of glucose, or during glycolysis inhibition, PMA induced chromatin decondensation but failed to induce NET release. Notably, the addition of glucose, but not pyruvate, was able to reinstate NETosis in these experiments[46]. NET production requires a shift in glucose metabolism to the PPP via glucose-6-phosphate dehydrogenase (G6PD)[60]. G6PD activity increases following PMA treatment via the action of PKC which phosphorylates serine and tyrosine residues neighbouring the active site of G6PD[61], and when G6PD activity is blocked NET production is abrogated. Glucose entering a neutrophil is phosphorylated by hexokinase to glucose-6-phosphate which can then flow through two pathways: PPP (producing NADPH and 6-phosphogluconolactone) or glycolysis (producing ATP and lactate). Production of NETs requires activation of the NADPH oxidase and production of ROS as well as mobilisation of granule enzymes such as elastase to the nucleus, leading to decondensation of nuclear chromatin prior to the release of extracellular DNA[3]. PMA has been shown to induce ROS production in glucose-free media[60]; however this was significantly lower than ROS produced in the presence of glucose. In the presence of a NOX2 inhibitor, PMA can induce low amounts of ROS, possibly *via* mitochondrial superoxide dismutase activity. However, mitochondrial ROS alone are not sufficient to trigger NET production[60], and neutrophils do not rely on mitochondrial function for NET release[46]. A number of amino acids and their intermediates were increased or decreased following PMA stimulation. This may represent a stimulation of transcription and translation in response to PMA[47], and/or the depletion of amino acids as glucose is re-directed from anaerobic glycolysis towards the PPP [56].

Our study is one of only two reporting global analysis of metabolites in human neutrophils. A recent study by Richer *et al*. [26] compared the metabolite profile of cytokine-treated neutrophils with that of untreated neutrophils and aged (30h) neutrophils. In this study, which did not include a heat-shock step, the authors report very low detection of transient metabolites NAD and NADPH in freshly isolated and cytokine-treated neutrophils, although high levels of NAD were detected in aged apoptotic neutrophils. Many of the other metabolites reported correlate well with the metabolites identified in our study, although they do not report the full list of annotated metabolites from their experiments, or state the number of annotated spectral bins.

## Conclusions

In summary, our results show that NMR metabolomics has great potential to identify functional changes in neutrophils activated *in vitro* or *in vivo* during inflammation. The metabolomics changes identified correlate well with the known and predicted changes in function of these cells and further work is now needed to perform these analyses on neutrophils stimulated *in vitro* with a wider range of agonists, and from patients with inflammatory disease, for example rheumatoid arthritis (RA). As has already been shown with transcriptomics[30, 41, 62], metabolomics has the potential to provide novel insight into disease heterogeneity, and could provide new information on the molecular effects of drug therapies on neutrophil function *in vivo*.

## Supporting information

S1 Fig Absence of Ficoll Paque in neutrophil NMR spectra. Green spectra shows neat Ficoll Paque. Black trace shows Ficoll Paque at expected maximum carry-through concentration in culture media. Neutrophil extracts shown for n = 3 experiments. No contaminating Ficoll Paque is visible in the neutrophil extracts.
(PDF)

S2 Fig Spectra showing NAD and NADP metabolite peaks. NAD and NADP peaks are barely detected above noise at 256NS, however peaks are clearly visible at 512NS and 2048NS.
(PDF)

S3 Fig Overlay of neutrophil metabolite extracts (n = 82). Insets show representative ^1^H NMR spectra for aliphatic and aromatic regions. Key metabolites are annotated as follows: (1) 1-Methylhistidine, (2) 3-Methylxanthine, (3) 4-Aminohippuric acid, (4) Acetic acid, (5) Adenine, (6) ADP, (7) D-Glucose, (8) D-Mannose, (9) Ethanol, (10) Formic acid, (11) Glycine, (12) Histamine, (13) Inosine, (14) L-Alanine, (15) L-Aspartic acid, (16) L-Isoleucine, (17) L-Lactic acid, (18) Lactulose, (19) Methylmalonic acid, (20) N-Acetyl-L-tyrosine, (21) N,N-Dimethylformamide, (22) NAD, (23) NADP, (24) NADPH, (25) Oxypurinol. Assignments detailed in S1 Table.
(PDF)

S4 Fig Change in neutrophil metabolome induced by PMA. Neutrophils were incubated in the presence of PMA (0.1 μg/mL) for 5 and 15 min (n = 3 biological replicates measured in technical triplicates). Supervised multivariate analysis by PLS-DA segregated 5 and 15 min samples (Q2 0.39248, R2 0.91143, accuracy 0.83333). Shading represents 95% confidence region. Scores plot is shown for components 1 and 2.
(PDF)

S5 Fig Change in neutrophil metabolome in untreated cells. Neutrophils were incubated without treatment for 5 and 15 min (n = 2 biological replicates measured in technical triplicates). Supervised multivariate analysis by PLS-DA was not able to segregate 5 and 15 min samples (Q2–6.4346, R2 0.93566, accuracy 0.16667). Shading represents 95% confidence region. Scores plot is shown for components 1 and 2.
(PDF)

S1 Table Metabolite assignments for S1 Fig. (PDF)

## References

[1] Glennon-Alty L, Hackett AP, Chapman EA, Wright HL. Neutrophils and redox stress in the pathogenesis of autoimmune disease. Free radical biology & medicine. 2018;125(25–35). 10.1016/j.freeradbiomed.2018.03.049 29605448

[2] Wright HL, Moots RJ, Bucknall RC, Edwards SW. Neutrophil function in inflammation and inflammatory diseases. Rheumatology (Oxford). 2010;49(9):1618–31. 20338884 10.1093/rheumatology/keq045

[3] Wright HL, Moots RJ, Edwards SW. The multifactorial role of neutrophils in rheumatoid arthritis. Nature reviews Rheumatology. 2014;10(10):593–601. 24914698 10.1038/nrrheum.2014.80

[4] Thieblemont N, Wright HL, Edwards SW, Witko-Sarsat V. Human neutrophils in auto-immunity. Seminars in immunology. 2016;28(2):159–73. 27036091 10.1016/j.smim.2016.03.004

[5] Denny MF, Yalavarthi S, Zhao W, Thacker SG, Anderson M, Sandy AR, et al A distinct subset of proinflammatory neutrophils isolated from patients with systemic lupus erythematosus induces vascular damage and synthesizes type I IFNs. Journal of Immunology. 2010;184(6):3284–97. 10.4049/jimmunol.0902199 PMC2929645 20164424

[6] O'Neill LA, Kishton RJ, Rathmell J. A guide to immunometabolism for immunologists. Nature reviews Immunology. 2016;16(9):553–65. 10.1038/nri.2016.70 27396447 PMC5001910

[7] Gaber T, Strehl C, Buttgereit F. Metabolic regulation of inflammation. Nat Rev Rheumatol. 2017;13(5):267–79. 28331208 10.1038/nrrheum.2017.37

[8] Weyand CM, Goronzy JJ. Immunometabolism in early and late stages of rheumatoid arthritis. Nat Rev Rheumatol. 2017;13(5):291–301. 28360422 10.1038/nrrheum.2017.49 PMC6820517

[9] Yang Z, Fujii H, Mohan SV, Goronzy JJ, Weyand CM. Phosphofructokinase deficiency impairs ATP generation, autophagy, and redox balance in rheumatoid arthritis T cells. The Journal of experimental medicine. 2013;210(10):2119–34. 24043759 10.1084/jem.20130252 PMC3782046

[10] Yang Z, Shen Y, Oishi H, Matteson EL, Tian L, Goronzy JJ, et al Restoring oxidant signaling suppresses proarthritogenic T cell effector functions in rheumatoid arthritis. Science translational medicine. 2016;8(331):331ra38. 10.1126/scitranslmed.aad7151 PMC5074090 27009267

[11] Emwas AH. The strengths and weaknesses of NMR spectroscopy and mass spectrometry with particular focus on metabolomics research. Methods in molecular biology. 2015;1277:161–93. 25677154 10.1007/978-1-4939-2377-9_13

[12] Lenz EM, Wilson ID. Analytical strategies in metabonomics. Journal of proteome research. 2007;6(2):443–58. 17269702 10.1021/pr0605217

[13] Beckonert O, Keun HC, Ebbels TM, Bundy J, Holmes E, Lindon JC, et al Metabolic profiling, metabolomic and metabonomic procedures for NMR spectroscopy of urine, plasma, serum and tissue extracts. Nature protocols. 2007;2(11):2692–703. 10.1038/nprot.2007.376 18007604

[14] Dunn WB, Ellis DI. Metabolomics: Current analytical platforms and methodologies. TrAC Trends in Analytical Chemistry. 2005;24(4):285–94.

[15] Hollywood K, Brison DR, Goodacre R. Metabolomics: current technologies and future trends. Proteomics. 2006;6(17):4716–23. 10.1002/pmic.200600106 16888765

[16] Lindon JC, Holmes E, Nicholson JK. So what's the deal with metabonomics? Analytical chemistry. 2003;75(17):384A–91A. 14632032 10.1021/ac031386+

[17] Corcoran O, Spraul M. LC-NMR-MS in drug discovery. Drug discovery today. 2003;8(14):624–31. 12867148 10.1016/s1359-6446(03)02749-1

[18] Dunn WB, Broadhurst DI, Atherton HJ, Goodacre R, Griffin JL. Systems level studies of mammalian metabolomes: the roles of mass spectrometry and nuclear magnetic resonance spectroscopy. Chemical Society reviews. 2011;40(1):387–426. 20717559 10.1039/b906712b

[19] Kapoor SR, Filer A, Fitzpatrick MA, Fisher BA, Taylor PC, Buckley CD, et al Metabolic profiling predicts response to anti-tumor necrosis factor alpha therapy in patients with rheumatoid arthritis. Arthritis and rheumatism. 2013;65(6):1448–56. 10.1002/art.37921 23460124 PMC3715109

[20] Young SP, Kapoor SR, Viant MR, Byrne JJ, Filer A, Buckley CD, et al The impact of inflammation on metabolomic profiles in patients with arthritis. Arthritis and rheumatism. 2013;65(8):2015–23. 10.1002/art.38021 23740368 PMC3840700

[21] Phelan MM, Caamano-Gutierrez E, Gant MS, Grosman RX, Madine J. Using an NMR metabolomics approach to investigate the pathogenicity of amyloid-beta and alpha-synuclein. Metabolomics: Official journal of the Metabolomic Society. 2017;13(12):151. 29142509 10.1007/s11306-017-1289-5 PMC5661010

[22] Thomas HB, Moots RJ, Edwards SW, Wright HL. Whose Gene Is It Anyway? The Effect of Preparation Purity on Neutrophil Transcriptome Studies. PLoS One. 2015;10(9):e0138982 10.1371/journal.pone.0138982 26401909 PMC4581699

[23] Calzetti F, Tamassia N, Arruda-Silva F, Gasperini S, Cassatella MA. The importance of being "pure" neutrophils. The Journal of allergy and clinical immunology. 2017;139(1):352–5 e6. 10.1016/j.jaci.2016.06.025 27567327

[24] Hallett MB, Lloyds D. Neutrophil priming: the cellular signals that say 'amber' but not 'green'. Immunol Today. 1995;16(6):264–8. 7662095 10.1016/0167-5699(95)80178-2

[25] Edwards SW. The O-2 Generating NADPH Oxidase of Phagocytes: Structure and Methods of Detection. Methods. 1996;9(3):563–77. 8812712 10.1006/meth.1996.0064

[26] Richer BC, Salei N, Laskay T, Seeger K. Changes in Neutrophil Metabolism upon Activation and Aging. Inflammation. 2018;41(2):710–21. 29322364 10.1007/s10753-017-0725-z

[27] Phelan M, Lian L. NMR Metabolomics: A Comparison of the Suitability of Various Commonly Used National Health Service Blood Collection Tubes. Current Metabolomics. 2016;4(1):78–81.

[28] Mitchell TS, Moots RJ, Wright HL. Janus kinase inhibitors prevent migration of rheumatoid arthritis neutrophils towards interleukin-8, but do not inhibit priming of the respiratory burst or reactive oxygen species production. Clinical and experimental immunology. 2017;189(2):250–8. 28369741 10.1111/cei.12970 PMC5508336

[29] Wright HL, Makki FA, Moots RJ, Edwards SW. Low-density granulocytes: functionally distinct, immature neutrophils in rheumatoid arthritis with altered properties and defective TNF signalling. Journal of leukocyte biology. 2017;101(2):599–611. 10.1189/jlb.5A0116-022R 27601627

[30] Wright HL, Cox T, Moots RJ, Edwards SW. Neutrophil biomarkers predict response to therapy with tumor necrosis factor inhibitors in rheumatoid arthritis. Journal of leukocyte biology. 2017;101(3):785–95. 27733572 10.1189/jlb.5A0616-258R

[31] Ammann C, Meier P, Merbach AE. A simple multinuclear NMR thermometer. Journal of Magnetic Resonance. 1982;46(2):319–21.

[32] Haug K, Salek RM, Conesa P, Hastings J, de Matos P, Rijnbeek M, et al MetaboLights—an open-access general-purpose repository for metabolomics studies and associated meta-data. Nucleic acids research. 2013;41(Database issue):D781–6. 23109552 10.1093/nar/gks1004 PMC3531110

[33] Sumner LW, Amberg A, Barrett D, Beale MH, Beger R, Daykin CA, et al Proposed minimum reporting standards for chemical analysis Chemical Analysis Working Group (CAWG) Metabolomics Standards Initiative (MSI). Metabolomics: Official journal of the Metabolomic Society. 2007;3(3):211–21. 24039616 10.1007/s11306-007-0082-2 PMC3772505

[34] Salek RM, Steinbeck C, Viant MR, Goodacre R, Dunn WB. The role of reporting standards for metabolite annotation and identification in metabolomic studies. GigaScience. 2013;2(1):13. 24131531 10.1186/2047-217X-2-13 PMC3853013

[35] Xia J, Sinelnikov IV, Han B, Wishart DS. MetaboAnalyst 3.0—making metabolomics more meaningful. Nucleic acids research. 2015;43(W1):W251–7. 10.1093/nar/gkv380 25897128 PMC4489235

[36] Xia J, Wishart DS. Using MetaboAnalyst 3.0 for Comprehensive Metabolomics Data Analysis. Current protocols in bioinformatics. 2016;55:14 0 1–0 91. 10.1002/cpbi.11 27603023

[37] Saeed AI, Bhagabati NK, Braisted JC, Liang W, Sharov V, Howe EA, et al TM4 microarray software suite. Methods in enzymology. 2006;411:134–93. 16939790 10.1016/S0076-6879(06)11009-5

[38] Hosak DA, Dennis G, Sherman BT, Lane HC, Lempicki RA. Identifying biological themes within lists of genes with EASE. Genome Biology. 2003;4(10):R70 10.1186/gb-2003-4-10-r70 14519205 PMC328459

[39] Berg JM, Tymoczko JL, Stryer L. Biochemistry: International Edition. 7th edition ed: W.H. Freeman, Basingstoke; 2012.

[40] Zhang D, Zhu M, Humphreys WG. Drug metabolism in drug design and development Basic Concepts and Practice: John Wiley & Sons; 2008.

[41] Wright HL, Thomas HB, Moots RJ, Edwards SW. RNA-Seq Reveals Activation of Both Common and Cytokine-Specific Pathways following Neutrophil Priming. PLoS One. 2013;8(3):e58598 10.1371/journal.pone.0058598 23554905 PMC3590155

[42] Maueroder C, Mahajan A, Paulus S, Gosswein S, Hahn J, Kienhofer D, et al Menage-a-Trois: The Ratio of Bicarbonate to CO2 and the pH Regulate the Capacity of Neutrophils to Form NETs. Frontiers in immunology. 2016;7:583. 28018350 10.3389/fimmu.2016.00583 PMC5145884

[43] Smolinska A, Attali A, Blanchet L, Ampt K, Tuinstra T, van Aken H, et al NMR and pattern recognition can distinguish neuroinflammation and peripheral inflammation. Journal of proteome research. 2011;10(10):4428–38. 21806074 10.1021/pr200203v

[44] Anderson JR, Chokesuwattanaskul S, Phelan MM, Welting TJM, Lian LY, Peffers MJ, et al (1)H NMR Metabolomics Identifies Underlying Inflammatory Pathology in Osteoarthritis and Rheumatoid Arthritis Synovial Joints. Journal of proteome research. 2018;17(11):3780–90. 10.1021/acs.jproteome.8b00455 30229649 PMC6220363

[45] Edwards SW. Biochemistry and physiology of the neutrophil: Cambridge University Press; 1994.

[46] Rodriguez-Espinosa O, Rojas-Espinosa O, Moreno-Altamirano MM, Lopez-Villegas EO, Sanchez-Garcia FJ. Metabolic requirements for neutrophil extracellular traps formation. Immunology. 2015;145(2):213–24. 25545227 10.1111/imm.12437 PMC4427386

[47] Khan MA, Palaniyar N. Transcriptional firing helps to drive NETosis. Scientific reports. 2017;7:41749 10.1038/srep41749 28176807 PMC5296899

[48] Borregaard N, Herlin T. Energy metabolism of human neutrophils during phagocytosis. The Journal of clinical investigation. 1982;70(3):550–7. 7107894 10.1172/JCI110647 PMC370256

[49] Stjernholm RL, Burns CP, Hohnadel JH. Carbohydrate metabolism by leukocytes. Enzyme. 1972;13:7–31. 4263081 10.1159/000459647

[50] Viant MR, Kurland IJ, Jones MR, Dunn WB. How close are we to complete annotation of metabolomes? Current opinion in chemical biology. 2017;36:64–9. 28113135 10.1016/j.cbpa.2017.01.001 PMC5337156

[51] Wishart DS, Feunang YD, Marcu A, Guo AC, Liang K, Vazquez-Fresno R, et al HMDB 4.0: the human metabolome database for 2018. Nucleic acids research. 2018;46(D1):D608–D17. 10.1093/nar/gkx1089 29140435 PMC5753273

[52] Wishart DS, Jewison T, Guo AC, Wilson M, Knox C, Liu Y, et al HMDB 3.0—The Human Metabolome Database in 2013. Nucleic acids research. 2013;41(Database issue):D801–7. 23161693 10.1093/nar/gks1065 PMC3531200

[53] Wishart DS, Knox C, Guo AC, Eisner R, Young N, Gautam B, et al HMDB: a knowledgebase for the human metabolome. Nucleic acids research. 2009;37(Database issue):D603–10. 10.1093/nar/gkn810 18953024 PMC2686599

[54] Wishart DS, Tzur D, Knox C, Eisner R, Guo AC, Young N, et al HMDB: the Human Metabolome Database. Nucleic acids research. 2007;35(Database issue):D521–6. 17202168 10.1093/nar/gkl923 PMC1899095

[55] Kramer PA, Ravi S, Chacko B, Johnson MS, Darley-Usmar VM. A review of the mitochondrial and glycolytic metabolism in human platelets and leukocytes: implications for their use as bioenergetic biomarkers. Redox biology. 2014;2:206–10. 24494194 10.1016/j.redox.2013.12.026 PMC3909784

[56] Loftus RM, Finlay DK. Immunometabolism: Cellular Metabolism Turns Immune Regulator. The Journal of biological chemistry. 2016;291(1):1–10. 26534957 10.1074/jbc.R115.693903 PMC4697146

[57] van Raam BJ, Sluiter W, de Wit E, Roos D, Verhoeven AJ, Kuijpers TW. Mitochondrial membrane potential in human neutrophils is maintained by complex III activity in the absence of supercomplex organisation. PLoS One. 2008;3(4):e2013 10.1371/journal.pone.0002013 18431494 PMC2295260

[58] Maianski NA, Geissler J, Srinivasula SM, Alnemri ES, Roos D, Kuijpers TW. Functional characterization of mitochondria in neutrophils: a role restricted to apoptosis. Cell death and differentiation. 2004;11(2):143–53. 10.1038/sj.cdd.4401320 14576767

[59] Fossati G, Moulding DA, Spiller DG, Moots RJ, White MR, Edwards SW. The mitochondrial network of human neutrophils: role in chemotaxis, phagocytosis, respiratory burst activation, and commitment to apoptosis. J Immunol. 2003;170(4):1964–72. 12574365 10.4049/jimmunol.170.4.1964

[60] Azevedo EP, Rochael NC, Guimaraes-Costa AB, de Souza-Vieira TS, Ganilho J, Saraiva EM, et al A Metabolic Shift toward Pentose Phosphate Pathway Is Necessary for Amyloid Fibril- and Phorbol 12-Myristate 13-Acetate-induced Neutrophil Extracellular Trap (NET) Formation. The Journal of biological chemistry. 2015;290(36):22174–83. 26198639 10.1074/jbc.M115.640094 PMC4571968

[61] Gupte RS, Ata H, Rawat D, Abe M, Taylor MS, Ochi R, et al Glucose-6-phosphate dehydrogenase is a regulator of vascular smooth muscle contraction. Antioxidants & redox signaling. 2011;14(4):543–58. E 20649491 10.1089/ars.2010.3207 PMC3029003

[62] Wright HL, Thomas HB, Moots RJ, Edwards SW. Interferon gene expression signature in rheumatoid arthritis neutrophils correlates with a good response to TNFi therapy. Rheumatology (Oxford, England). 2015;54(1):188–93. 25125592 10.1093/rheumatology/keu299

