# Supplementary figures and images for: A robust intracellular metabolite extraction protocol for human neutrophil metabolic profiling
Source: PLoS One. 2018 Dec 20;13(12):e0209270. doi: 10.1371/journal.pone.0209270 (PMC6301625; doi:10.1371/journal.pone.0209270)

Neutrophil extracts (x3)

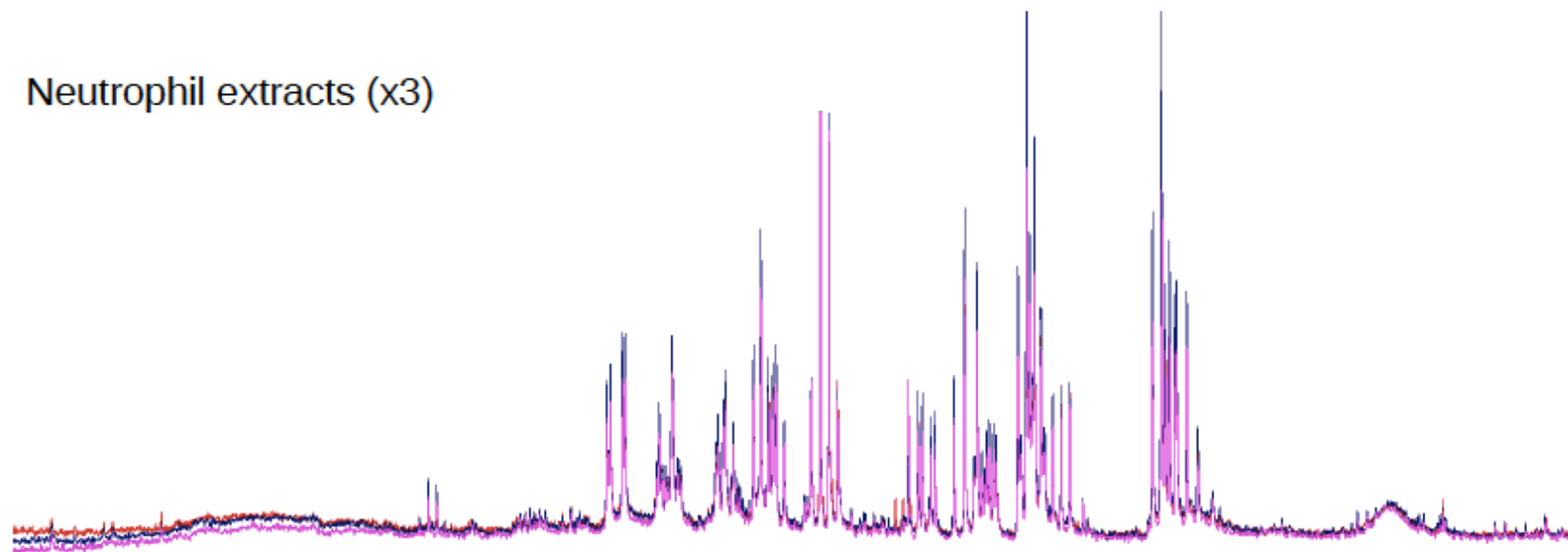

Ficoll-paque

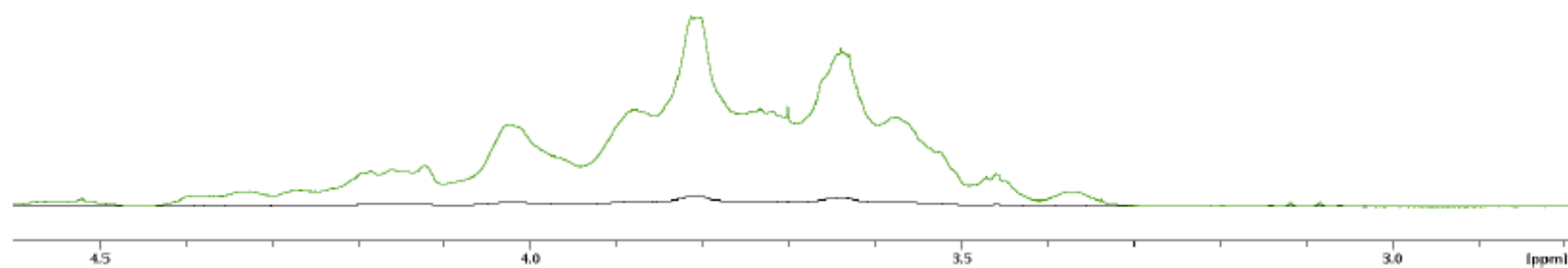

Supplement: S1 Fig — Green spectra shows neat Ficoll Paque. Black trace shows Ficoll Paque at expected maximum carry-through concentration in culture media. Neutrophil extracts shown for n = 3 experiments. No contaminating Ficoll Paque is visible in the neutrophil extracts. (PDF) [file pone.0209270.s001.pdf]

NS 2048

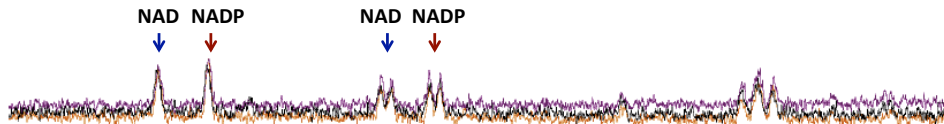

NS 512

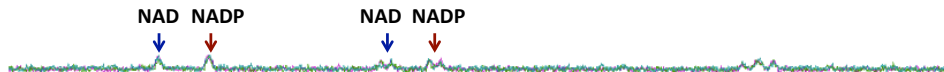

NS 256

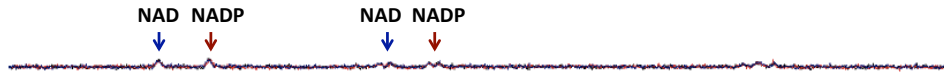

9.4

9.2

9.0

8.8

[ppm]

Supplement: S2 Fig — NAD and NADP peaks are barely detected above noise at 256NS, however peaks are clearly visible at 512NS and 2048NS. (PDF) [file pone.0209270.s002.pdf]

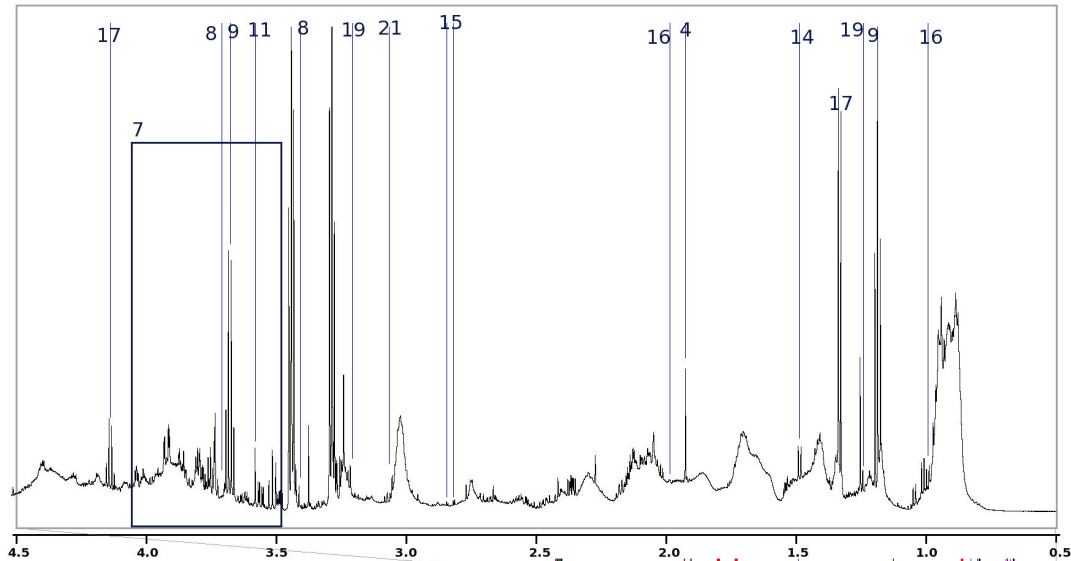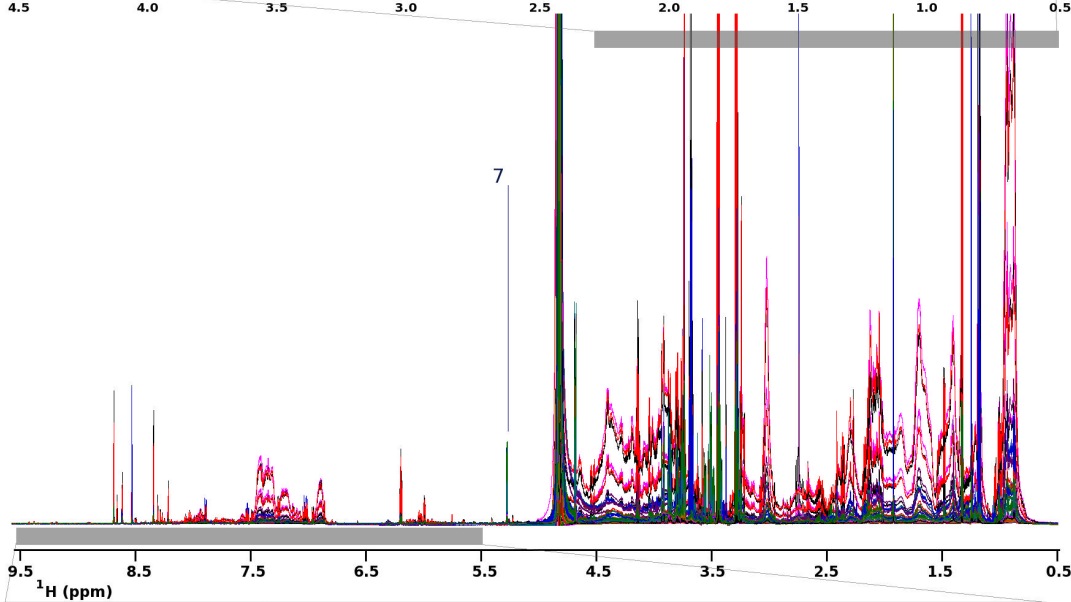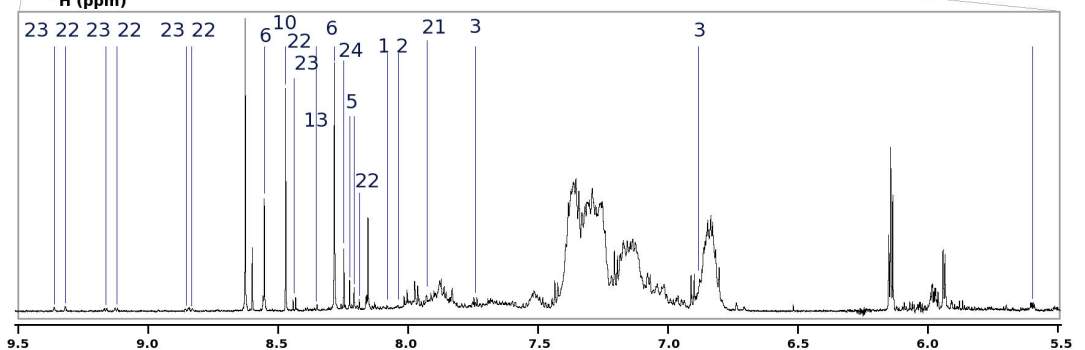

Supplement: S3 Fig — Insets show representative 1H NMR spectra for aliphatic and aromatic regions. Key metabolites are annotated as follows: (1) 1-Methylhistidine, (2) 3-Methylxanthine, (3) 4-Aminohippuric acid, (4) Acetic acid, (5) Adenine, (6) ADP, (7) D-Glucose, (8) D-Mannose, (9) Ethanol, (10) Formic acid, (11) Glycine, (12) Histamine, (13) Inosine, (14) L-Alanine, (15) L-Aspartic acid, (16) L-Isoleucine, (17) L-Lactic acid, (18) Lactulose, (19) Methylmalonic acid, (20) N-Acetyl-L-tyrosine, (21) N,N-Dimethylformamide, (22) NAD, (23) NADP, (24) NADPH, (25) Oxypurinol. Assignments detailed in S1 Table. (PDF) [file pone.0209270.s003.pdf]

Scores Plot

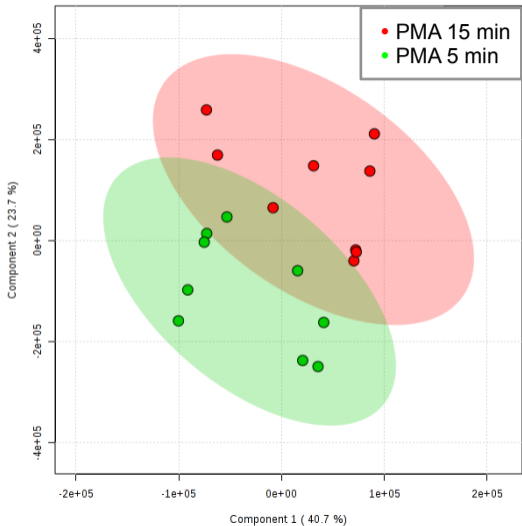

Supplement: S4 Fig — Neutrophils were incubated in the presence of PMA (0.1 μg/mL) for 5 and 15 min (n = 3 biological replicates measured in technical triplicates). Supervised multivariate analysis by PLS-DA segregated 5 and 15 min samples (Q2 0.39248, R2 0.91143, accuracy 0.83333). Shading represents 95% confidence region. Scores plot is shown for components 1 and 2. (PDF) [file pone.0209270.s004.pdf]

Scores Plot

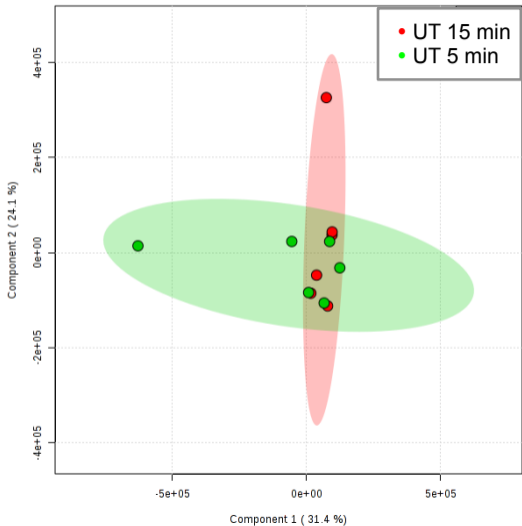

Supplement: S5 Fig — Neutrophils were incubated without treatment for 5 and 15 min (n = 2 biological replicates measured in technical triplicates). Supervised multivariate analysis by PLS-DA was not able to segregate 5 and 15 min samples (Q2–6.4346, R2 0.93566, accuracy 0.16667). Shading represents 95% confidence region. Scores plot is shown for components 1 and 2. (PDF) [file pone.0209270.s005.pdf]
