# Supplementary material for: A robust intracellular metabolite extraction protocol for human neutrophil metabolic profiling
Source: PLoS One. 2018 Dec 20;13(12):e0209270. doi: 10.1371/journal.pone.0209270 (PMC6301625; doi:10.1371/journal.pone.0209270)
Supplement: S1 Table — (PDF) [file pone.0209270.s006.pdf]

S1 Table: Metabolite assignments for S1 Fig

Database identifier	Metabolite identification	Supplementary Figure 1 ID	Chemical shift*
HMDB0000001	1-Methylhistidine	1	[8.0761...8.0673]
HMDB0000034	Adenine	5	[8.2252...8.2112]
HMDB0000042	Acetic acid	4	[1.9263...1.9161]
HMDB0000108	Ethanol	9	[3.6854...3.6775], [3.6775...3.6698], [3.6620...3.6548], [3.6492...3.6380], [1.2008...1.1838], [1.1801...1.1740]
HMDB0000122	D-Glucose	7	[5.2545...5.2278], [3.9173...3.8852], [3.8568...3.8188], [3.7523...3.7397], [3.7175...3.7031], [3.5583...3.5484], [3.5417...3.5358], [3.5336...3.5288], [3.5037...3.4933], [3.4886...3.4740], [3.4740...3.4612], [3.4110...3.4067], [3.4067...3.3999], [3.2554...3.2495]
HMDB0000123	Glycine	11	[3.5665...3.5583]
HMDB0000142	Formic acid	10	[8.4716...8.4436]
HMDB0000161	L-Alanine	14	[1.4941...1.4735]
HMDB0000169	D-Mannose	7	[3.9311...3.9229], [3.8779...3.8568], [3.7755...3.7626], [3.3999...3.3634]
HMDB0000172	L-Isoleucine	16	[2.0051...1.9655], [1.0268...1.0071], [0.9622...0.9527]
HMDB0000190	L-Lactic acid	17	[4.1337...4.1059], [4.1059...4.0862], [1.3436...1.3196]
HMDB0000191	L-Aspartic acid	15	[3.9229...3.9173], [2.8349...2.8244], [2.8106...2.7995], [2.7053...2.7001], [2.6846...2.6771]
HMDB0000195	Inosine	13	[8.3566...8.3459]
HMDB0000202	Methylmalonic acid	19	[3.1935...3.1852], [3.1815...3.1661], [1.2485...1.2383]
HMDB0000217	NADP	23	[9.3190...9.2929], [8.8231...8.8091], [8.4272...8.4083], [8.2046...8.1923], [6.1234...6.1162]
HMDB0000221	NADPH	24	[8.4921...8.4773], [8.2555...8.2457], [6.9754...6.9528], [4.0862...4.0574], [4.0574...4.0400], [2.8645...2.8349], [2.7995...2.7735]
HMDB0000740	Lactulose	18	[4.0245...4.0059], [3.9584...3.9359], [3.9359...3.9311], [3.8188...3.8084], [3.7031...3.6854], [3.6312...3.6226], [3.5842...3.5665]
HMDB0000786	Oxypurinol	25	[8.2876...8.2572]
HMDB0000866	N-Acetyl-L-tyrosine	20	[7.7924...7.7686], [6.8601...6.8514], [6.8514...6.8428], [3.1110...3.0856], [1.9367...1.9330]
HMDB0000870	Histamine	12	[7.9291...7.9169], [7.1414...7.1308], [3.3160...3.3111], [3.0371...3.0267]

HMDB0000902	NAD	22	[9.3513...9.3190], [9.1599...9.1266], [8.8478...8.8379], [8.8379...8.8231], [8.4387...8.4272], [8.1734...8.1488]
HMDB0001341	ADP	6	[8.5679...8.5182], [6.1557...6.1405]
HMDB0001867	4-Aminohippuric acid	3	[8.3278...8.3057], [7.6917...7.6848], [7.6848...7.6751], [7.6751...7.6732]
HMDB0001886	3-Methylxanthine	2	[8.0341...8.0277], [3.5358...3.5336]
HMDB0001888	N,N-Dimethylformamide	21	[7.9412...7.9371], [3.0442...3.0371]

*where peak overlap occurs only 1 representative metabolite listed. Full assignment (including overlapped peaks available online via Metabolights ID MTBLS658)
